# Brief Glutamine Pretreatment Increases Alveolar Macrophage CD163/Heme Oxygenase-1/p38-MAPK Dephosphorylation Pathway and Decreases Capillary Damage but Not Neutrophil Recruitment in IL-1/LPS-Insufflated Rats

**DOI:** 10.1371/journal.pone.0130764

**Published:** 2015-07-06

**Authors:** Ana Fernandez-Bustamante, Amanda Agazio, Paul Wilson, Nancy Elkins, Luke Domaleski, Qianbin He, Kaily A. Baer, Angela F. D. Moss, Paul E. Wischmeyer, John E. Repine

**Affiliations:** 1 Department of Anesthesiology, University of Colorado SOM, Aurora, Colorado, United States of America; 2 Department of Medicine, University of Colorado SOM, Aurora, Colorado, United States of America; 3 Webb-Waring Center, University of Colorado SOM, Aurora, Colorado, United States of America; 4 Adult and Child Center for Health Outcomes and Delivery Science (ACCORDS), University of Colorado SOM, Aurora, Colorado, United States of America; Chinese Academy of Sciences, CHINA

## Abstract

**Background:**

Glutamine (GLN) attenuates acute lung injury (ALI) but its effect on alveolar macrophages is unknown. We hypothesized that GLN pretreatment would induce the anti-inflammatory CD163/heme oxygenase (HO)-1/p38-MAPK dephosphorylation pathway in alveolar macrophages and reduce ALI in rats insufflated with interleukin-1 (IL-1) and lipopolysaccharide (LPS).

**Methods:**

Male Sprague-Dawley rats were randomized to the following groups: GLN-IL-1/LPS-, GLN+IL-1/LPS-, GLN-IL-1/LPS+, and GLN+IL-1/LPS+. GLN pretreatment was given via gavage (1g/kg L-alanyl-L-glutamine) daily for 2 days. ALI was subsequently induced by insufflating 50ng IL-1 followed by 5mg/kg *E*.*coli* LPS. After 24h, bronchoalveolar lavage (BAL) protein, lactate dehydrogenase (LDH) and neutrophil concentrations were analyzed. BAL alveolar macrophage CD163+ expression, HO-1 and p38-MAPK concentrations were measured, as well as alveolar macrophage tumor necrosis factor (TNF)-α and interleukin (IL)-10 concentrations. Histology and immunofluorescence studies were also performed.

**Results:**

Following IL-1/LPS insufflation, GLN pretreated rats had significantly decreased BAL protein and LDH concentrations, but not BAL neutrophil counts, compared to non-GLN pretreated rats. The number of alveolar macrophages and the number of CD163+ macrophages were significantly increased in GLN pretreated IL-1/LPS-insufflated rats compared to non-GLN pretreated, IL-1/LPS-insufflated rats. GLN pretreatment before IL-1/LPS also significantly increased HO-1 concentrations and dephosphorylated p38-MAPK levels but not cytokine levels in alveolar macrophages. Immunofluorescence localized CD163 and HO-1 in alveolar macrophages.

**Conclusion:**

Short-term GLN pretreatment activates the anti-inflammatory CD163/HO-1/p38-MAPK dephosphorylation pathway of alveolar macrophages and decreases capillary damage but not neutrophil recruitment in IL-1/LPS-insufflated rats.

## Introduction

Glutamine (GLN) is a critical amino acid that is highly utilized by activated lymphocytes and macrophages[1–4]. In addition, GLN administration decreases acute lung injury (ALI) in animal models where lymphocytes and macrophages are activated[5–15]. Previously, Oliveira *et al*.[5] observed that intravenous GLN administration increased the number of alveolar macrophages in septic malnourished rats. Because sepsis and other episodes involving intense immune responses (i.e., burns, major surgery) are associated with decreased plasma levels of GLN[16–19], a relative GLN deficiency could impair the function of macrophages and lymphocytes and contribute to ALI development (now known clinically as the Acute Respiratory Distress Syndrome, ARDS).

Nonetheless, no studies have specifically analyzed the effect of GLN supplementation on alveolar macrophages during ALI development. This is surprising since alveolar macrophages are key elements during the insult, resolution, and repair phases of ALI[20–23]. Indeed, the extremely versatile alveolar macrophages appear to adjust their phenotype and function depending not only on the stage of the inflammatory process but also the type of insult[24, 25]. For example, during the injury phase, alveolar macrophage pro-inflammatory mechanisms can release chemotaxins that recruit damaging neutrophils into the lung[26]. It is known that alveolar macrophage depletion significantly increases neutrophil recruitment[27, 28] in sepsis or lipopolysaccharide (LPS)-induced animal models of ALI. During the repair phase, alveolar macrophage anti-inflammatory mechanisms may decrease ALI and initiate other beneficial processes.

The exact mechanisms that might contribute to alveolar macrophage attenuating ALI remain to be elucidated. We focused herein on the possibility that inducing alveolar macrophage CD163/heme oxygenase-1 (HO-1)/p38-MAPK dephosphorylation may contribute to ALI reduction. HO-1 is a well-known inducible enzyme that metabolizes pro-oxidant heme groups into less injurious biliverdin (later transformed to bilirubin), carbon monoxide and iron. Induction of the HO-1 enzyme is regulated primarily by the presence of heme substrate, a response that happens often during capillary damage in LPS and other insult-generated ALI models. Heme groups, by up-regulating the surface expression of macrophage-specific CD163, also known as hemoglobin scavenger receptor, induce HO-1 in macrophages [29–31]. The CD163 macrophage receptor is increasingly attracting attention in part because of its emerging potential as therapeutic target for activating macrophage anti-inflammatory features [32, 33]. HO-1 induction also has well-known anti-inflammatory features through its effect on several cell types that could attenuate ALI [34–37] One of these potentially protective mechanisms is the dephosphorylation and deactivation of the p38-MAPK pathway in macrophages and other cell types[35, 38, 39].

GLN is a non-specific inducer of heat shock proteins (HSP), including HSP32 or HO-1, during oxidative stress insults[40, 41]. Although a non-specific HO-1 inducer, using GLN as a potential therapy is attractive because of its established high oral and enteral uptake and high safety profile in animals and humans[42–44]. Moreover, specific HO-1 inducers (i.e. CoPP) can successfully up-regulate HO-1 *in vitro*, in animal models *in vivo* following an intraperitoneal injection[37], and in humans (i.e. hemin) when given intravenously[45].

In the present investigation, we found that GLN supplementation activates the anti-inflammatory CD163/HO-1/p38-MAPK dephosphorylation pathway in alveolar macrophages and prevents ALI features related to capillary damage in rats insufflated with IL-1/LPS.

## Methods

### Ethics statement

All experimental protocols were approved by the University of Colorado Institutional Animal Care and Use Committee (IACUC) (approval #21912(01)1E).

### Experimental animal model

Sprague-Dawley (350±50g) rats were randomized to one of the following four groups (at least 8 rats per group): GLN-IL-1/LPS-, GLN+IL-1/LPS-, GLN-IL-1/LPS+, and GLN+IL-1/LPS+. Glutamine (GLN) treated rats were gavaged with 1g/kg L-alanyl-L-glutamine (ALA-GLN) dipeptide dissolved in 2 mL drinking water daily for 2 consecutive days (total 2 doses) to increase L-GLN availability. ALA-GLN was used as it is stable in solution (unlike L-GLN) [46]. 1 g of ALA-GLN dipeptide provides 0.75 g of L-GLN[43]. 24h after the second GLN dose, rats were anesthetized with pentobarbital (50mg/kg intraperitoneal, IP). After cleaning the neck skin, the trachea was exposed through a small incision of the ventral neck. ALI was induced by instilling interleukin-1 (IL-1) and lipopolysaccharide (LPS) intratracheally as described[47]. Briefly, a single dose of 50ng of recombinant IL-1 (R&D Systems #200-LA-002/CF) diluted in 0.5mL saline was insufflated intratracheally using a 24-gauge IV catheter that was immediately removed. An hour later, another bolus of 5mg/kg *Escherichia coli* LPS 0111:B4 (Sigma-Aldrich #L2630) in 0.5mL saline was insufflated intratracheally. After the second IT bolus, the neck incision was sutured closed and rats were allowed to spontaneously breath and recover from anesthesia. No tracheal instrumentation and/or insufflation was done to control rats. Previous experiments evaluating tracheal insufflation of 0.5mL saline did not change the bronchoalveolar total cellular content, differential (i.e. macrophage, neutrophil) cell counts or acute lung injury features (i.e. BAL LDH concentration) compared to non-insufflated rats (minimum of 8 animals per group). Accordingly, non-insufflated rats were used as controls for this study. After 24h, all rats were again anesthetized with IP pentobarbital, the neck incision reopened and a tracheotomy was performed. A laparotomy and thoracotomy were performed. A left lower lung lobe was tied off to avoid contamination and lung disturbance during instillation of lavage fluid, and used for histology and immunohistochemistry studies. The remaining lungs were lavaged with a total 17mL of saline via the tracheotomy. Bronchoalveolar lavage fluid (BAL) was then immediately recovered and saved for analysis. One lung was then excised and quick-frozen in liquid nitrogen while the other lung was formaldehyde fixed for histological and immunofluorescence analyses. Blood was obtained from the inferior vena cava and plasma samples were collected in heparinized vials, aliquoted and frozen at -70 degrees. No mechanical ventilation was performed during this approximately 2-minute process.

### Acute Lung injury features

#### BAL total protein concentration

The protein concentration of BAL samples was quantified using the bicinchoninic acid (BCA) method. Briefly, supernatants from cell-free BAL cell lysates were measured by colorimetry and read against a BCA protein standard curve (Sigma-Aldrich Cat# P0914, 1 mg bovine serum albumin/ml in 0.15 M NaCl). The average of sample triplicates from each BAL sample was used for statistical analysis.

#### BAL lactate dehydrogenase (LDH)

LDH was measured in the recovered BAL fluid samples using the Promega CytoTox 96 Non-Radioactive Cytotoxicity Assay (Promega Corporation Cat#G1780, purchased from Fisher Scientific, Houston, TX).

#### BAL total cell counts and differential counts

Total cell and leukocyte counts were determined using a hemocytometer. Wright-stained cytospin preparations of BAL samples were used to obtain differential macrophage and neutrophil counts as previously described[48].

#### Lung histology

Lung sections were formalin-fixed for 24 hours, transferred to 70% alcohol and paraffin-embedded. Paraffin blocks were cut with a microtome, de-waxed and stained with hematoxylin & eosin (H&E). Representative images were examined blindly for each group and photographed with a Nikon Eclipse 6600 microscope. Representative images from different rats from each group were blindly assessed by 3 co-authors and graded for neutrophil content and location (alveolar and interstitial), septal thickening, proteinaceous debris, and hyaline membranes using the lung injury scoring system recommended by the American Thoracic Society (ATS)[49] (briefly, the formula recommended by the ATS can be summarized as: ALI Score = [(20 × alveolar neutrophils) + (14 × interstitial neutrophils) + (7 × hyaline membranes) + (7 × proteinaceous debris) + (2 × alveolar septal thickening)]/(number of fields × 100)]. Please see full details in reference[49]).

### Alveolar macrophage assays

#### Macrophage isolation

Alveolar macrophages were isolated from BAL samples by centrifugation at 2,400 rpm for 10 minutes at 4°C. Supernatants were saved for later analysis and cells were resuspended in 1 mL RPMI-1640 media without L-glutamine and phenol red (Sigma-Aldrich R5709) but supplemented with 10% charcoal stripped Fetal Bovine Serum (Gibco 12676–011), and 1% Penicillin Streptomycin Solution (HyClone SV30010). Subsequently, 200 μL of resuspended cells were plated with 800 μL of media in a 12 well flat bottom tissue culture plate (Corning Life Sciences DL 353043). The cells were allowed to adhere for 1.5 hours at 37°C with 5% CO_2_. Once the incubation was complete, media and non-adherent cells were aspirated and Cell Lysis Buffer containing phosphatase inhibitors (1mM activated Sodium Orthovanadate, 20mM Sodium Fluoride, 2.5mM Sodium Pyrophosphate) was added to each well. The tissue culture plate was placed on ice for 30 minutes under slight agitation before the wells were scrapped. Next, cell lysates were aliquoted into 1.5 mL microcentrifuge tubes, vortexed, and chilled on ice for five minutes. Cell lysates were vortexed again, centrifuged at 13,000 rpm for 15 minutes at 4°C and stored at -80°C until used.

#### Heme oxygenase-1 (HO-1), p38-MAPK, TNF-α and IL-10 concentrations

Concentrations in the macrophage lysates were analyzed following manufacturers’ instructions for HO-1 (Enzo, cat# ADI-EKS-810A), total p38-MAPK (Invitrogen, cat# KHO0061), phosphorylated p38-MAPK (p38 [pTpY180/182, Invitrogen, cat# KHO0071), TNF-α (RayBio, cat# ELR-TNFa-CL) and IL-10 (RayBio, cat# ELR-IL10-CL) by ELISA in duplicate samples. Dephosphorylated p38-MAPK was calculated by subtracting phosphorylated p38-MAPK from the total p38-MAPK concentrations for each rat.

#### Quantification of CD163+ macrophages using flow-cytometry

Directly conjugated primary antibody APC-CD163 (Bioss Inc., Woburn, MA) was applied to 5 x 10^4^ BAL cells in FACS running buffer (0.5%BSA and 2mM EDTA in PBS) and incubated in the dark for 15 minutes at room temperature on an orbital shaker. Cells were rinsed two times with FACS running buffer and flow cytometry measurements were performed using a seven color MacsQuant Analyzer (Miltenyi Biotec). Fluorescence was compared to unstained controls for 35,000 events in a histogram display, and the stained APC-CD163 positive events were compensated for unstained events by subtraction. Data analysis was performed using FCS Express 4 Flow Cytometry De Novo Software.

#### Immunofluorescence for CD163 and HO-1

To visualize the cellular source and co-localization of HO-1 and CD163, paraffin-embedded lung slices were rehydrated and permeabilized in PBS with 0.3% Triton X-100. Lung sections were then incubated sequentially in PBS solutions containing 10% donkey serum at room temperature for 1 h, then with anti-rabbit HO-1 (Enzo, cat#ADI-EKS-810A) and anti-mouse CD163 (Abd Serotec, cat#MCA342R) in a 2% BSA solution overnight at 4°C. Following primary antibody incubation, tissue sections were washed and incubated with pre-adsorbed donkey F(ab)anti mouse IgG(H&L, cat#ab150103) conjugated with Alexa-Fluor-647 (donkey Fab2 Rabbit-IgG-HL-Alexa-Fluor647) and donkey F(ab)anti mouse IgG (H&L, cat# ab175694) conjugated with Alexa-Fluor-568 (donkey Fab2 Rabbit-IgG-HL-Alexa-Fluor568), respectively, for 1h at room temperature in a dark hydration chamber. Tissue sections were then washed, stained with ProLong Gold Antifade Reagent with DAPI, Invitrogen, cat#P36935), covered, and stored at 4°C. Images were captured using an Olympus FV-1000 confocal microscope.

### Statistical analysis

All measurements are shown as mean ± SEM unless otherwise indicated. A two-way ANOVA was conducted to examine the effect of glutamine pre-treatment and IL-1/LPS insufflation on each continuous variable. When glutamine and IL-1/LPS had a statistically significant interaction, this is reported. Tukey p values were used for pairwise comparisons between the groups. A *p*<0.05 was considered to be statistically significant. Statistical analyses were performed with SAS 9.4 software.

## Results

### Acute lung injury assessments

#### BAL total protein concentrations

**Fig 1:**
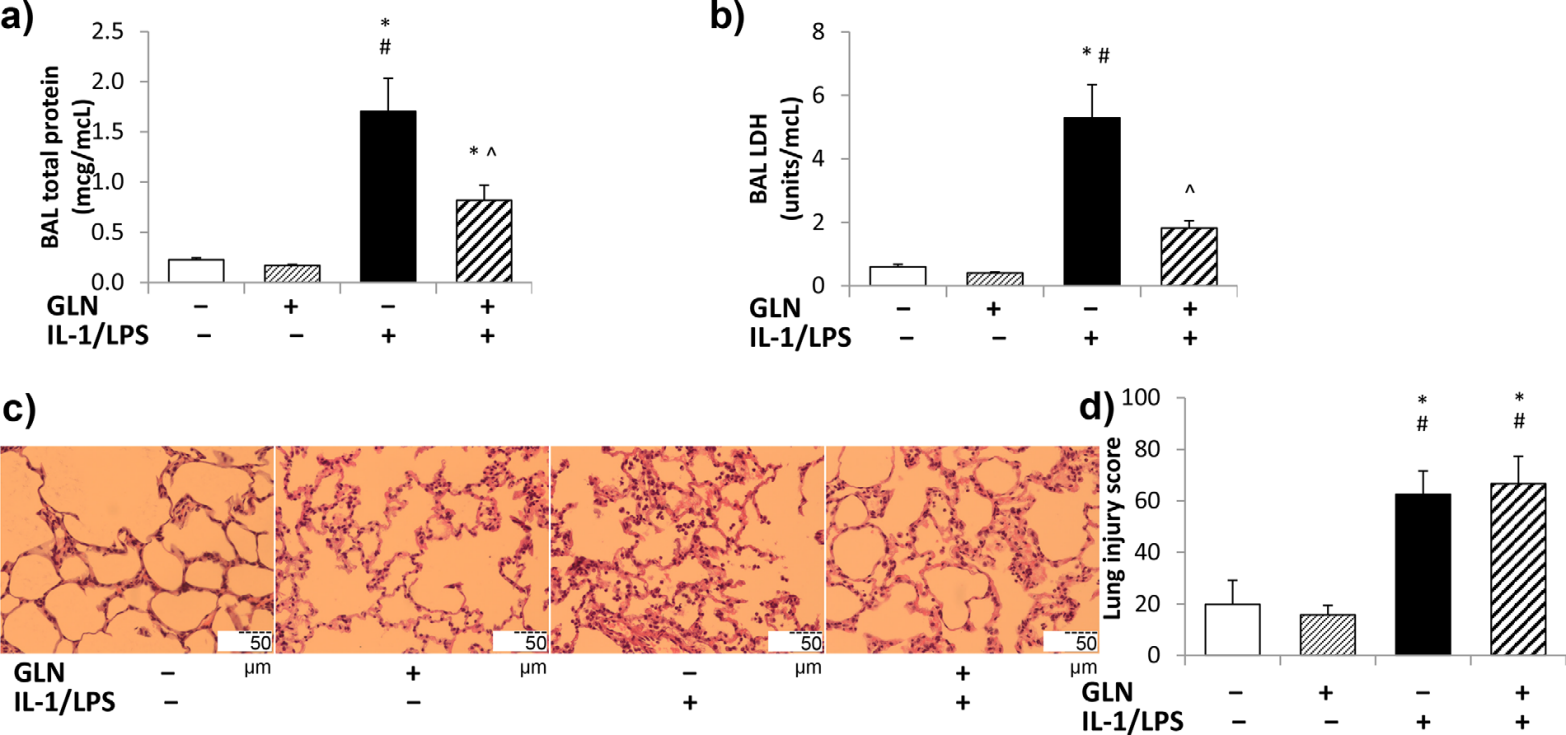
Lung injury features. There was no interaction between GLN pretreatment and IL-1/LPS insufflation on BAL protein concentrations (1a). IL-1/LPS significantly increased BAL protein levels in both GLN+ and GLN- groups. GLN+IL-1/LPS+ groups had significantly lower BAL protein concentrations than GLN-IL-1/LPS+ groups. GLN and IL-1/LPS showed a significant interaction on BAL LDH concentration (1b). BAL LDH concentrations were significantly higher in GLN-IL-1/LPS+ groups compared to both IL-1/LPS- groups. GLN+IL-1/LPS+ rats had significantly lower BAL LDH levels than GLN-IL-1/LPS+ groups. Fig 1c shows representative H&E histology images from each group. IL-1/LPS insufflation increased the blindly assessed histological lung injury scores with no interaction or effect by GLN pretreatment (1d). (The significance of differences among the four groups was analyzed by two-way ANOVA followed by paired comparisons. Statistical significance was accepted as p<0.05: * compared to GLN-IL-1/LPS- group, # compared to respective GLN+IL-1/LPS- group, ^ compared to GLN-IL-1/LPS+ group).

No interaction was found between GLN and IL-1/LPS on BAL protein levels. BAL protein concentrations were similar in both non-IL-1/LPS groups (Fig 1a). Following IL-1/LPS, BAL protein concentrations significantly increased compared to non-GLN non-IL-1/LPS rats, but BAL protein levels in the GLN-pretreated IL-1/LPS-insufflated group were significantly lower than in the non-GLN IL-1/LPS-insufflated group.

#### BAL LDH concentrations

GLN and IL-1/LPS showed a significant interaction on BAL LDH concentrations. BAL LDH concentrations were similar in both non-IL-1/LPS groups (Fig 1b). IL-1/LPS insufflation increased the BAL LDH concentrations compared to the non-IL-1/LPS insufflated groups, but this increase was statistically significant only in the non-GLN group and not the GLN-pretreated group. BAL LDH levels in GLN-pretreated IL-1/LPS-insufflated rats were significantly lower than in non-GLN IL-1/LPS-insufflated rats.

#### Lung histology

Fig 1c shows representative hematoxylin & eosin stained histology slides from lungs of the 4 rat groups. Lung parenchymal walls exhibited a normal architecture in non-IL-1/LPS insufflated rats. In contrast, in both GLN pretreated and untreated rats following IL-1/LPS insufflation, lungs had thickened irregular walls with increased cellular infiltration. Histological injury changes in the GLN pretreated IL-1/LPS insufflated rats were less homogeneously distributed than in the untreated ones, but the lung injury scores performed by three blinded investigators revealed no detectable effect found by GLN pretreatment.

#### BAL total cell numbers and differential counts

**Fig 2:**
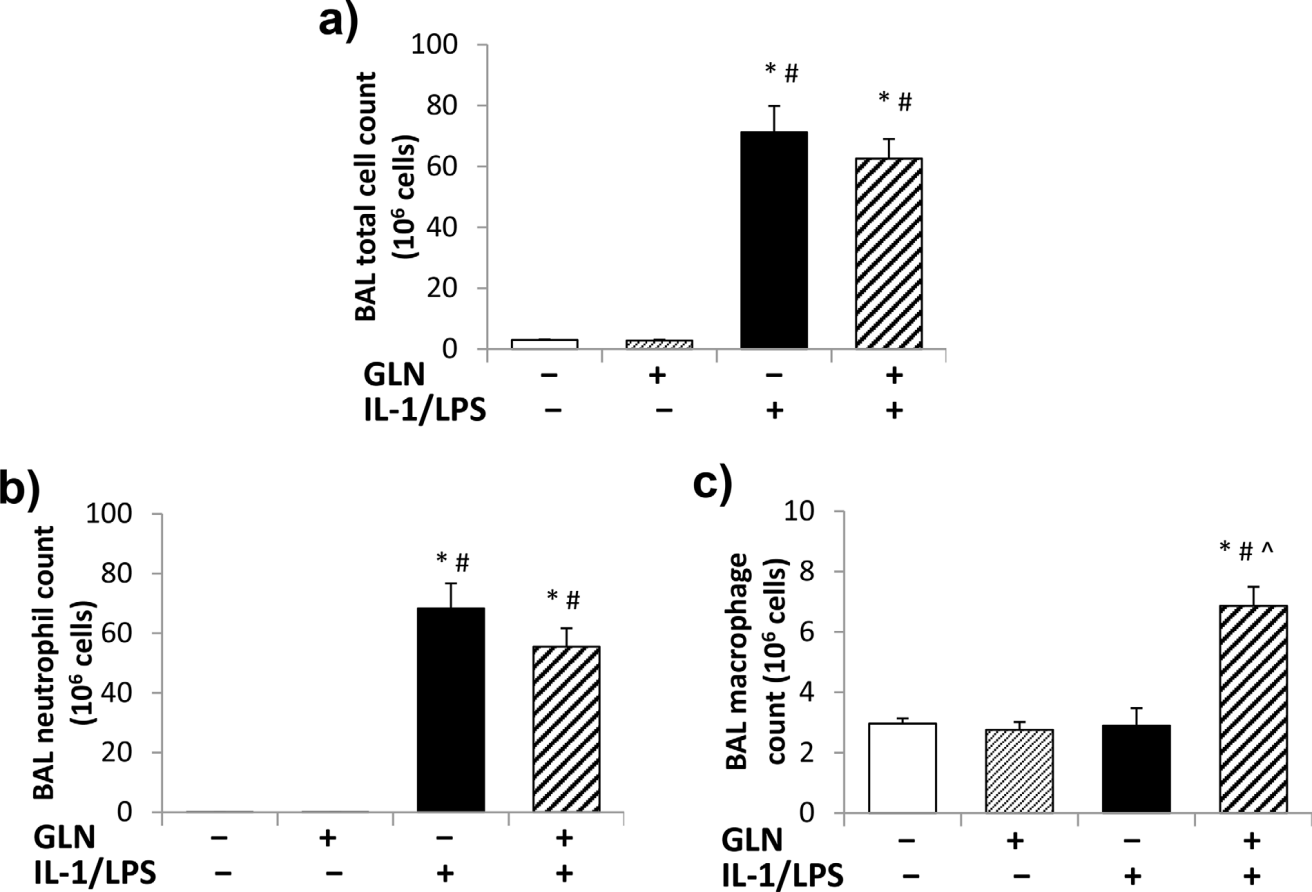
Total BAL cell numbers and cell differential counts. IL-1/LPS significantly increased the BAL total cell numbers without a significant effect by GLN pretreatment (2a). No interaction between GLN and IL-1/LPS was observed on BAL neutrophil numbers (2b). BAL neutrophils significantly increased with IL-1/LPS insufflation but were not significantly affected by GLN pretreatment. Conversely, GLN and IL-1/LPS showed a significant interaction on BAL macrophage numbers (2c) with both GLN and IL-1/LPS significantly increasing the numbers of BAL macrophages in the GLN+IL-1/LPS+ group compared to the other three groups. (The significance of differences among the four groups was analyzed by two-way ANOVA. See statistical methods section for details on paired comparisons. Statistical significance was accepted as p<0.05: * compared to GLN-IL-1/LPS- group, # compared to respective GLN+IL-1/LPS- group, ^ compared to GLN-IL-1/LPS+ group).

The total cell numbers in BAL samples from non-IL-1/LPS groups with or without GLN pretreatment were (Fig 2a). Following IL-1/LPS insufflation, BAL total cell numbers were significantly increased compared to non-IL-1/LPS groups (Fig 1a). No significant differences were found in the BAL total cell numbers of IL-1/LPS-insufflated rats with or without GLN pretreatment. However, the similar BAL total cell numbers of IL-1/LPS-insufflated rats, with and without GLN pretreatment, included a shift in BAL cell differential (neutrophil/macrophage) distribution. An overall shift to decreased neutrophils and increased macrophages was observed in that GLN-pretreated IL-1/LPS-insufflated rats had 86.9±1.2% alveolar macrophages and 13.1±1.2% neutrophils while in contrast non-GLN IL-1/LPS-insufflated rats had 95.1±0.9% alveolar macrophages and 4.9±0.9% neutrophils. While these changes were not statistically different, the absolute differences accounted for the changes in macrophage and neutrophil numbers.

BAL neutrophil counts were similar in both non-IL-1/LPS groups (Fig 2b). IL-1/LPS insufflation significantly increased BAL neutrophil counts in both groups. However, BAL neutrophil counts were statistically not different in GLN-pretreated IL-1/LPS-insufflated rats (55.7±6.2 million) than in non-GLN IL-1/LPS-insufflated rats (68.3±8.4 million) (p = 0.214). No significant interaction or effect was observed by GLN on BAL neutrophil counts. Conversely, GLN and IL-1/LPS showed a significant interaction on BAL macrophage counts (Fig 2c). BAL macrophage counts were significantly higher in GLN-pretreated IL-1/LPS-insufflated rats (6.9±0.6 million) compared to non-GLN non-IL-1/LPS (3.0±0.2 million), GLN-pretreated non-IL-1/LPS (2.8±0.3 million), and non-GLN IL-1/LPS-insufflated (2.9±0.6 million) rats.

### Alveolar macrophage assessments

#### Quantification of CD163+ macrophages using flow-cytometry

**Fig 3:**
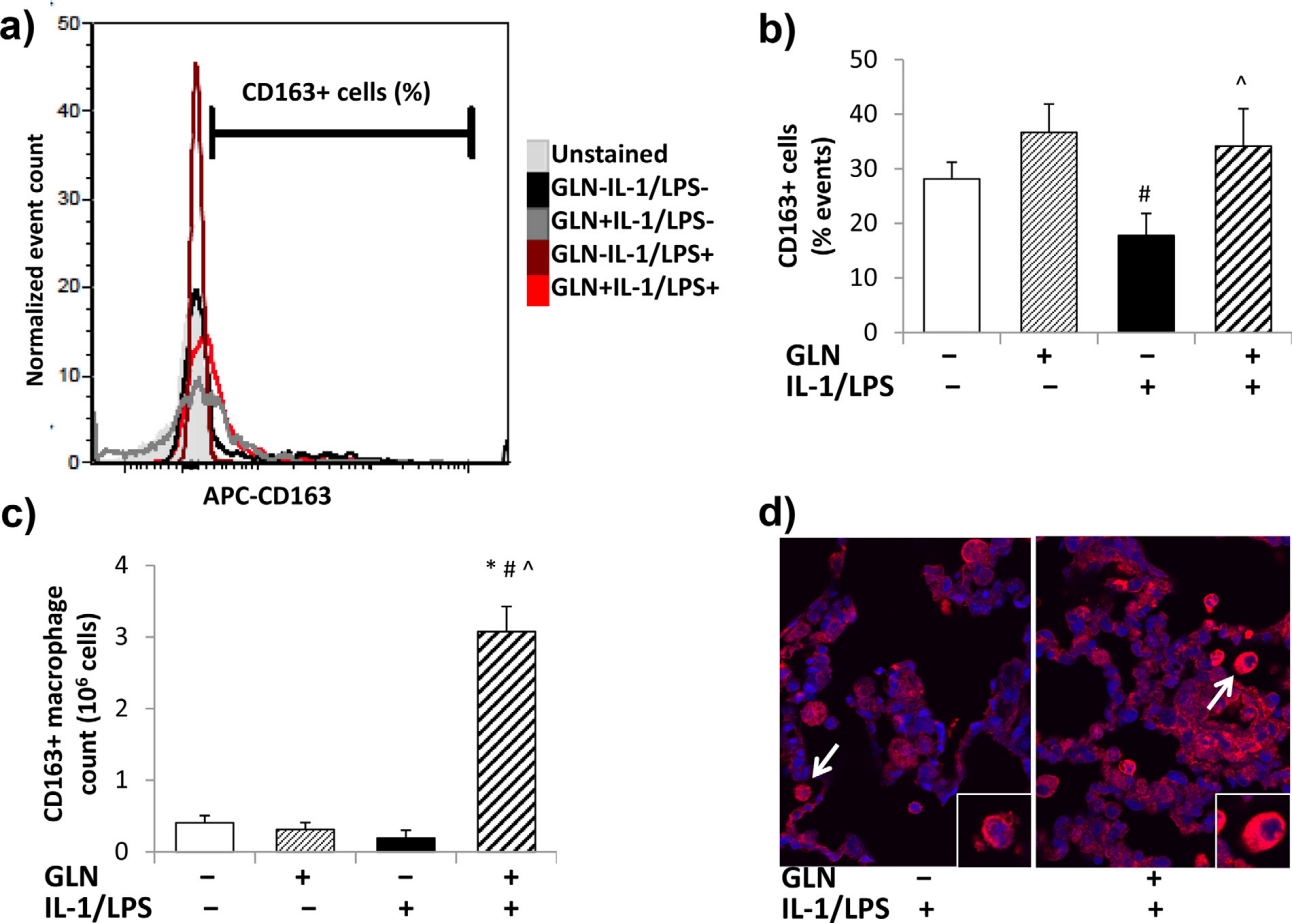
Quantification of CD163+ BAL cells by flow-cytometry. Fig (**3a**) shows representative flow-cytometry histograms from studied groups. Fluorescence was compared to unstained controls for 35,000 events in each histogram display, and the stained APC-CD163 positive events were compensated for unstained events by subtraction. A 5% error margin was accepted in the unstained image. GLN and IL-1/LPS showed no interaction on the percentage of CD163+ events (**3b**). The percentage of CD163+ events was significantly increased by GLN but not affected by IL-1/LPS. GLN and IL-1/LPS significantly interacted on the number of CD163+ macrophages (**3c**). CD163+ macrophage numbers were significantly increased in GLN+IL-1/LPS+ rats by both GLN and IL-1/LPS. Fig **3d** shows immunofluorescence representation of CD163 stained lung regions from the GLN-IL-1/LPS+ and GLN+IL-1/LPS+ groups (white arrow points to zoomed cell in bottom right corner) (DAPI nuclear staining = blue; CD163 = red). (The significance of differences among the four groups was analyzed by two-way ANOVA. See statistical methods section for details on paired comparisons. Statistical significance was accepted as p<0.05: * compared to respective GLN- group, # compared to respective IL-1/LPS- group, ^ significant interaction between GLN and IL-1/LPS group).

Fig 3a depicts a representative flow-cytometry histogram of each of the 4 groups. No significant interaction was found between GLN pretreatment and IL-1/LPS insufflation on the percentage of CD163+ macrophages in the reconstituted BAL cell pellet. GLN pretreatment significantly increased the percentage of CD163+ macrophages without any significant effect induced by IL-1/LPS (Fig 3b). This percentage of CD163+ macrophages was multiplied by the total BAL macrophage count to calculate the CD163+ macrophage counts for each rat (Fig 3c). Both GLN and IL-1/LPS significantly interacted on the observed increased CD163+ macrophage counts in the GLN-pretreated IL-1/LPS-insufflated group compared to the other three groups. Immunofluorescence located CD163 staining in alveolar macrophages (Fig 3d).

#### Heme oxygenase-1 concentration in isolated macrophages

**Fig 4:**
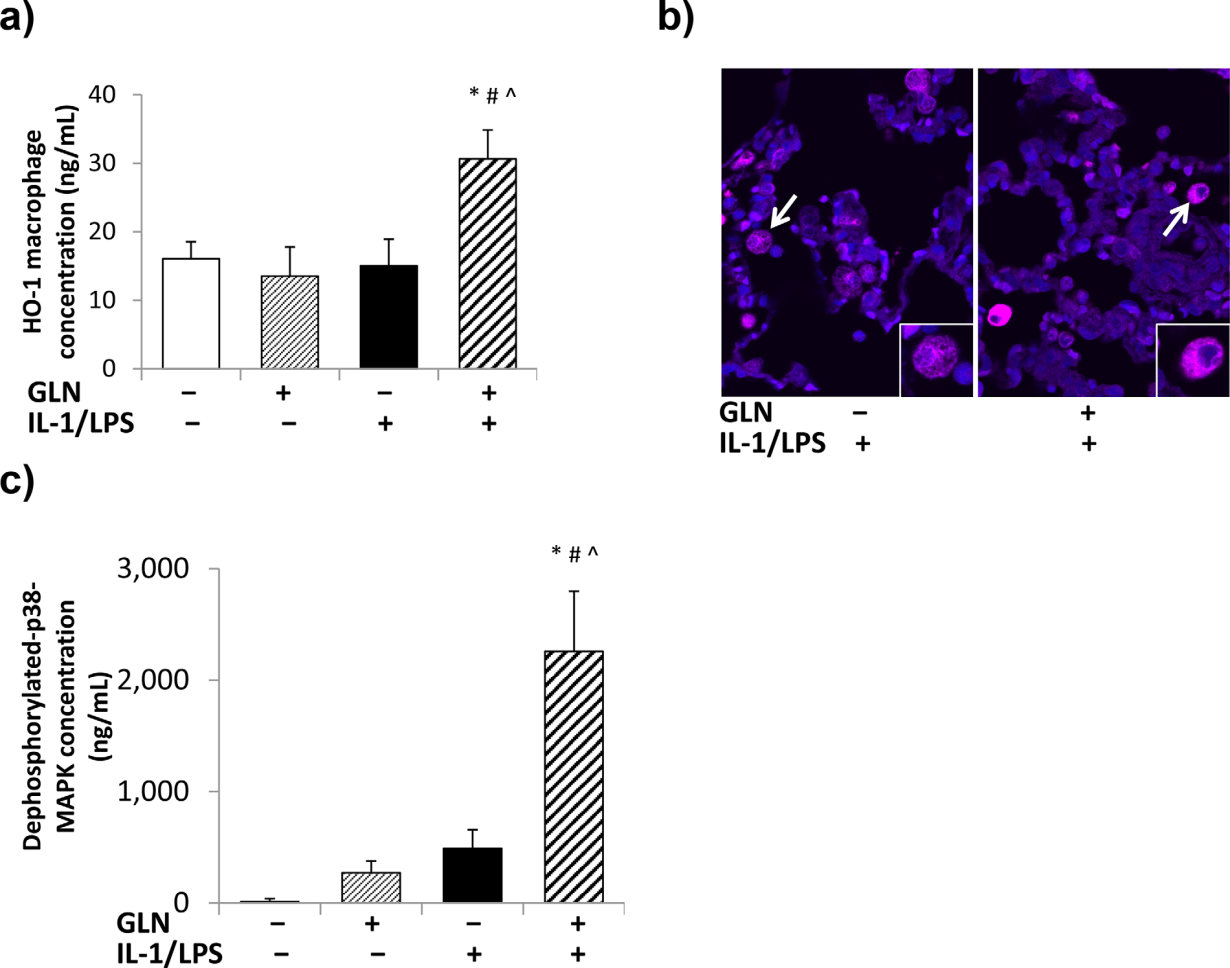
Measurements of HO-1 and dephosphorylated p38-MAPK concentrations in alveolar macrophages. The concentration of HO-1 in isolated alveolar macrophages (**4a**) was significantly increased exclusively by IL-1/LPS with no interaction or effect of GLN pretreatment. Fig **4b** shows immunofluorescence representation of HO-1 stained lung regions from GLN-IL-1/LPS+ and GLN+IL-1/LPS+ groups. (white arrow points to zoomed cell in bottom right corner) (DAPI nuclear staining = blue; HO-1 = pink). Dephosphorylated p38-MAPK concentrations (**4c**) were significantly increased in the GLN+IL-1/LPS+ group compared to the other three groups, and both GLN and IL-1/LPS had a significantly interaction on this increase. (The significance of differences among the four groups was analyzed by two-way ANOVA. See statistical methods section for details on paired comparisons. Statistical significance was accepted as p<0.05: * compared to respective GLN- group, # compared to respective IL-1/LPS- group, ^ significant interaction between GLN and IL-1/LPS group).

GLN and IL-1/LPS showed a significant interaction on macrophage HO-1 concentrations. Paired comparisons showed significantly higher macrophage HO-1 concentrations in GLN-pretreated IL-1/LPS-insufflated rats compared to both non-IL-1/LPS groups and the non-GLN IL-1/LPS-insufflated group (Fig 4a). Immunofluorescence located HO-1 staining in alveolar macrophages (Fig 4b).

#### Measurements of dephosphorylated p38-MAPK, TNF-α and IL-10 levels in isolated alveolar macrophages

Both GLN and IL-1/LPS showed a significant interaction with respect to macrophage dephosphorylated p38-MAPK levels. Alveolar macrophages from GLN-pretreated IL-1/LPS-insufflated rats had significantly increased dephosphorylated p38-MAPK levels compared to both non-IL-1/LPS rats and the non-GLN IL-1/LPS-insufflated rats (Fig 4c). No statistically significant changes were observed in macrophage cytokine concentrations between the 4 rat groups.

## Discussion

We observed that a brief pretreatment with ALA-GLN dipeptide (“glutamine, GLN”) significantly decreased ALI as reflected by decreases in BAL protein and LDH concentrations in rats insufflated with IL-1/LPS. Glutamine pretreatment before IL-1/LPS insufflation increased the number of alveolar macrophages and activated the alveolar macrophage anti-inflammatory CD163/HO-1/p38-MAPK dephosphorylation pathways, but did not significantly decrease neutrophil recruitment into the lung. These changes were not observed when GLN pretreatment and/or IL-1/LPS insufflation were administered separately.

Different GLN treatment modalities have successfully attenuated ALI in animal models. The approaches have been both preventative (if given before or simultaneously with the inciting insult)[14] and therapeutic (given after the insult and during the establishing injury)[5–7]. For example, treatment with a 10-day course of 1g/kg GLN by gavage not only reduced histologic features of ALI but also decreased serum TNF-α, IL-6 and IL-10 concentrations in Sprague-Dawley rats given oleic acid[14]. A daily dose of 0.75g/kg of ALA-GLN gavaged, starting after LPS insufflation, reduced lung interstitial inflammation and levels of pro-inflammatory and fibrosis-related biomarkers in mice[7]. In addition, administering GLN decreased ALI induced by sepsis or LPS[5–9], ischemia-reperfusion[10, 11], hyperoxia[12, 13], oleic acid[14] and smoke inhalation in multiple animal models[15].

Surprisingly, while GLN administration decreased ALI, it did not, however, decrease the number of neutrophils recruited into the lung or the histological injury (heavily influenced by neutrophil infiltration). The reason why glutamine treatment did not decrease neutrophil recruitment into the lung following IL-1/LPS insufflation is unknown but could be related to insufficient GLN pretreatment (dose or duration) and/or other mechanisms. Nonetheless, our finding suggests that the GLN-dependent injury prevention is not likely a direct effect of GLN on neutrophils but rather on alveolar macrophages or other cell types. Significantly, glutamine pretreatment prevented ALI despite the presence of large numbers of recruited neutrophils.

We confirmed that GLN pretreatment changed alveolar macrophages mostly after IL-1/LPS insufflation. A significant increase in the number of alveolar macrophages occurred in GLN pretreated IL-1/LPS insufflated rats, but not following GLN pretreatment or IL-1/LPS insufflation individually. GLN treatment is known to increase the number of alveolar macrophages[5], but the responsible mechanism is unclear. The possibilities include glutamine treatment reducing apoptosis of macrophages, enhancing recruitment of interstitial macrophages released from the lung parenchyma, and/or an accelerating recruitment of peripheral blood monocytes. Oliveira *et at*.[5] showed a decreased caspase-3-related apoptosis in lung macrophages after intravenous GLN treatment in malnourished rats both before and after cecal ligation and puncture (CPL)-induced sepsis.

We also found that dephosphorylated p38-MAPK, with known anti-apoptotic features[38], was significantly increased in macrophages of GLN pretreated injured rats. The significance of this change remains to be determined but it may have contributed to the increased number of macrophages in GLN pretreated rats after IL-1/LPS. Detachment of alveolar macrophages from adjacent epithelial cells is another possibility. This mechanism has been described as a feature of the complex role that alveolar macrophages exhibit during quick defensive responses to pathogens[50]. Recruitment of blood monocytes into an inflammatory site in a LPS-induced murine peritonitis model occurs rapidly, often within 1.5–2 hours and independently from neutrophil activation and/or neutrophil-derived increase in MCP-1[51]. Henderson *et al.[51]* concluded that resident (peritoneal) macrophages may release MCP-1 directly or in response to a TNF-α increase, leading to the quick recruitment of blood monocytes after a peritoneal LPS insult. The significance of this concept needs additional investigation since recent findings suggest that peritoneal alveolar macrophages differ in several aspects from lung interstitial macrophages[50]. This difference in macrophages may also impact the sophisticated intercommunication role played by macrophages with respect to causing and modulating neutrophil recruitment into the lung[28, 52].

We also found that the alveolar macrophages of GLN pretreated rats developed multiple anti-inflammatory features including increased CD163 expression, HO-1 content, and dephosphorylated P38-MAPK concentrations and confirmed by immunofluorescence the localization of these GLN triggered responses in alveolar macrophages. The CD163/HO-1 pathway is a relevant anti-inflammatory mechanism in macrophages. CD163 belongs to the scavenger receptor cysteine-rich super family (SRCR-SF) class B, and is activated primarily by increased free heme [30, 31, 53]. CD163 is located exclusively in the outer membrane of certain monocyte/macrophages including alveolar macrophages and other mature tissue macrophages[33]. The main function of CD163 is acting as a hemoglobin transporter by endocytosis, promoting heme clearance, and reducing oxidative damage. Notably, LPS and other pro-inflammatory processes downregulate CD163 expression[31, 53].

We observed a trend to fewer CD163+ macrophages in untreated rats receiving IL-1/LPS insufflation (Fig 3), and significantly increased CD163+ macrophages in GLN pretreated rats insufflated with IL-1/LPS. CD163+ monocyte/macrophages increase during the resolution of inflammation and manifest increased HO-1 synthesis^28,29^. HO-1 expression was also increased in macrophages from GLN pretreated IL-1/LPS-insufflated rats. HO-1 has well-known anti-inflammatory effects in macrophages and in ALI[31, 35, 37, 54–57]. Several studies have reported that HO-1 related anti-inflammatory and anti-apoptotic effects occur via the p38-MAPK dephosphorylation or inactivation[35, 38, 58, 59], which was also apparent in alveolar macrophages from GLN pretreated injured rats. The involvement of the p38-MAPK pathway may also contribute to protection conferred by GLN against heat stress in intestinal epithelial cells *in vitro*[39].

GLN is a frequently used nutraceutical supplement that can act as a non-selective inducer of heat shock proteins (HSP), including HSP32 or heme oxygenase-1 (HO-1). GLN treatment increases HSP-70 or HSF-1 in animals exposed to LPS or sepsis [60, 61] but this effect has not always been confirmed [55]. HO-1 has also been successfully induced by GLN treatment in animal models of liver ischemia-reperfusion (I/R) *in vivo*[62] and in anoxia/reoxygenation of human proximal renal tubular epithelial cells (HK-2) *in vitro*[59]. Since we focused on the macrophage CD163/HO-1 pathway, we did not study other HSPs and, accordingly, cannot exclude a possible protective effect on ALI as a consequence of GLN-mediated changes in the non-CD163-related induction of other HSPs.

Several studies have reported that GLN has a critical effect on modulating macrophage activation and, more specifically, contributes to the balance between arginine/nitric oxide production and glutamate nucleotide synthesis[1–4]. GLN consumption is increased during macrophage activation[2–4]. GLN concentrations in plasma are significantly reduced during episodes of intense catabolic stress, such as in response to major surgery, critical illness, and intense exercise[16–19]. More specifically, a recent metabolomics study by Bordbar *et al*.[1] highlighted a key role of GLN during macrophage activation in response to LPS[1]. This concept could explain our finding of why GLN protected against ALI when IL-1/LPS was insufflated but had only minimal effects (increased proportion of CD163+ macrophages) in the absence of IL-1/LPS. It is possible that GLN pretreatment prevented a relative macrophage deficiency that develops during ALI and in response to the IL-1/LPS insult. Glutamine may have achieved this effect by switching macrophages to a more repair-oriented phenotype.

Additionally, GLN treatment changes T cells[7]. Hou *et al*.[7] recently observed reduced ALI in mice administered a single intratracheal bolus of LPS followed by daily gavage of GLN for up to 10 days. In the latter study, the protective effect by GLN was associated with an increase in regulatory T cells and IL-2, and a down-regulation of T helper 17 cells. Hu *et al*.[6] also found that administering GLN IV increased the number of immunoregulatory gammadelta T cells in the lungs and decreased neutrophil infiltration in septic control mice after cecal ligation and puncture (CLP)-induced sepsis.

Our study was not designed to establish a cause-effect relationship between GLN administration, the observed CD163/HO-1/p38-MAPK dephosphorylation changes in alveolar macrophages, and ALI development following IL-1/LPS insufflation. Furthermore, an insufficient total dose and/or the timing (24h before injury induction and 48h before lung injury analyses) of GLN administration may have accounted for the lack of significant reduction in neutrophil recruitment. More prolonged treatment with GLN in several animal models consistently has shown a greater benefit (including decreased neutrophil infiltration). We selected this short regimen as a potentially feasible approach with a clinically relevant preventative application: administration of a short supplementation of glutamine immediately before a foreseeable injury, such as an elective surgical procedure with risk of postoperative infection or early on in the suspected risk of developing ALI. Although the lack of tracheal instrumentation in our control rats may pose some concerns as the ideal control for injured rats, in our preliminary experiments, as discussed in the methods section, this factor did not show significant differences in critical lung injury features The timing of GLN administration in relation to ALI (and the type of insult, LPS *vs*. others) may be critical, and despite its proven safety profile[44], might explain the variable success observed in human GLN studies[42, 43, 63].

## Conclusions

Brief gavaging of ALA-GLN dipeptide for two consecutive days beforehand significantly decreased bronchoalveolar protein and LDH concentrations, increased the number of alveolar macrophages, and increased the CD163/HO-1/p-38-MAPK dephosphorylation pathway in alveolar macrophages of rats insufflated with IL-1/LPS. Additional studies are warranted to further understand the exact effect of GLN on alveolar macrophage function and its relationship to ALI development. Evaluating the relationship between GLN supplementation dosing and timing in different types of insults (LPS-mediated *vs*. others) may clarify the potential of this relatively safe nutritional supplement in animals and humans.

## Supporting Information

S1 File The Minimal Data Set contains data used in this study. (PDF)
