# Supplementary material for: Brief Glutamine Pretreatment Increases Alveolar Macrophage CD163/Heme Oxygenase-1/p38-MAPK Dephosphorylation Pathway and Decreases Capillary Damage but Not Neutrophil Recruitment in IL-1/LPS-Insufflated Rats
Source: PLoS One. 2015 Jul 6;10(7):e0130764. doi: 10.1371/journal.pone.0130764 (PMC4493112; doi:10.1371/journal.pone.0130764)
Supplement: S1 File — (PDF) [file pone.0130764.s001.pdf]

	Gln	IL1.LPS	cellcount	pmn.coun	macs.cou nt	pmn.perc ent	macs.perc ent	prot	ldh	mac.ho1	mac.tnfa	mac.IL10
1	0	0	2.83	.01	2.82	.33	99.67	.21	.48	.	.	.
2	0	0	3.65	.05	3.60	1.33	98.67	.31	1.25	.	.	.
3	0	0	4.48	.15	4.33	3.33	96.67	.14	.29	.	.	.
4	0	0	3.05	.01	3.04	.33	99.67	.19	.42	.	.	.
5	0	0	2.98	.01	2.97	.33	99.67	.17	.33	.	.	.
6	0	0	2.60	.03	2.57	1.33	98.67	.21	.58	.	.	.
7	0	0	2.63	.02	2.61	.67	99.33	.21	.43	.	.	.
8	0	0	3.68	.02	3.66	.67	99.33	.	.	7.01	.	.
9	0	0	3.48	.02	3.46	.67	99.33	.	.	17.32	.	.
10	0	0	4.20	.00	4.20	.00	100.00	.	.	24.47	.	.
11	0	0	1.35	.00	1.35	.00	100.00	.	.	15.41	.	.
12	0	0	1.35	.03	1.32	2.00	98.00	.13	.40	.	.	.
13	0	0	3.98	.04	3.94	1.00	99.00	.13	.43	.	.	.
14	0	0	3.38	.06	3.32	1.67	98.33	.12	.21	.	.	.
15	0	0	3.53	.00	3.49	.07	99.00	.11	.42	.	.	.
16	0	0	2.40	.01	2.37	.33	98.67	.10	.35	.	.	.
17	0	0	2.78	.00	2.78	.00	100.00	.	.	.	.	690.55
18	0	0	1.95	.03	1.92	1.33	98.67	.	.	.	.	.
19	0	0	2.08	.01	2.06	.67	99.33	.	.	.	.	.
20	0	0	2.90	.02	2.88	.67	99.33	.21	.48	.	.	.
21	0	0	2.85	.00	2.85	.00	100.00	.19	1.17	.	.	.
22	0	0	1.40	.03	1.37	2.00	98.00	.20	1.18	.	.	.
23	0	0	4.48	.03	4.45	.67	99.33	.15	.53	.	.	.
24	0	0	4.78	.06	4.71	1.33	98.67	.22	.98	28.09	.	747.03
25	0	0	3.85	.03	3.82	.67	99.33	.23	.88	25.34	.	872.82
26	0	0	4.78	.02	4.76	.33	99.67	.15	.	.	.	862.79
27	0	0	3.45	.00	3.45	.00	100.00	.10	.	.	.	.
28	0	0	2.65	.15	2.50	5.67	94.33	.28	.	.	.	.
29	0	0	2.13	.00	2.13	.00	100.00	.43	.	.	.	.
30	0	0	3.50	.02	3.48	.67	99.33	.30	.	.	.	.
31	0	0	4.37	.04	4.32	1.00	99.00	.30	.	.	.	.
32	0	0	2.45	.00	2.45	.00	100.00	.23	.	16.17	.	.
33	0	0	1.95	.00	1.95	.00	100.00	.17	.	11.23	.	.
34	0	0	2.43	.02	2.41	.67	99.33	.20	.	9.40	.	.
35	0	0	1.78	.00	1.78	.00	100.00	.15	.	6.08	.	.
36	0	0	3.50	.02	3.48	.67	99.33	.49	.	.	.	.
37	0	0	1.95	.00	1.95	.00	100.00	.33	.	.	.	.
38	0	0	1.96	.04	1.92	2.00	98.00	.59	.	.	.	.
39	0	0	2.59	.02	2.57	.67	99.33	.37	.	.	.	.

	mac.p38mapk	mac.Pp38mapk	CD163.percent	cd163.count	lung.injury.score	pmn.alv	pmn.interst	hyaline.mbms	prot.debris	septal.thickng
1	.	.	24.68	.70	2.0	.0	.0	.0	?	?
2	.	.	31.98	1.15	4.0	.0	.0	.0	.0	2.0
3	.	.	20.38	.88	53.0	.0	2.0	1.0	2.0	2.0
4	.	.	23.87	.73	.0	.0	.0	.0	.0	.0
5	83.94	129.65	48.89	1.45	16.0	.0	.0	.0	2.0	1.0
6	123.68	125.19	37.81	.97	44.0	.0	1.0	2.0	2.0	1.0
7	254.79	166.48	24.78	.65	.	.	.	.	.	.
8	.	.	.	.	.	.	.	.	.	.
9	.	.	.	.	.	.	.	.	.	.
10	.	.	.	.	.	.	.	.	.	.
11	.	.	.	.	.	.	.	.	.	.
12	.	.	.	.	.	.	.	.	.	.
13	.	.	.	.	.	.	.	.	.	.
14	.	.	.	.	.	.	.	.	.	.
15	.	.	.	.	.	.	.	.	.	.
16	.	.	.	.	.	.	.	.	.	.
17	138.54	134.12	.	.	.	.	.	.	.	.
18	.	.	.	.	.	.	.	.	.	.
19	.	.	.	.	.	.	.	.	.	.
20	.	.	20.06	.58	.	.	.	.	.	.
21	.	.	4.42	.13	.	.	.	.	.	.
22	.	.	.	.	.	.	.	.	.	.
23	.	.	25.53	1.13	.	.	.	.	.	.
24	.	.	.	.	.	.	.	.	.	.
25	.	.	.	.	.	.	.	.	.	.
26	.	.	.	.	.	.	.	.	.	.
27	.	.	.	.	.	.	.	.	.	.
28	.	.	.	.	.	.	.	.	.	.
29	.	.	.	.	.	.	.	.	.	.
30	.	.	.	.	.	.	.	.	.	.
31	.	.	.	.	.	.	.	.	.	.
32	.	.	.	.	.	.	.	.	.	.
33	.	.	.	.	.	.	.	.	.	.
34	.	.	.	.	.	.	.	.	.	.
35	.	.	.	.	.	.	.	.	.	.
36	.	.	.	.	.	.	.	.	.	.
37	.	.	.	.	.	.	.	.	.	.
38	.	.	.	.	.	.	.	.	.	.
39	.	.	.	.	.	.	.	.	.	.

	Gln	IL1.LPS	cellcount	pmn.coun	macs.count	pmn.percent	macs.perc ent	prot	ldh	mac.ho1	mac.tnfa	mac.IL10
40	0	0	.	.	.	.	.	.20	.	.	.	.
41	?	0	.	.	.	.	.	.22	.	.	.	.
42	?	0	.	.	.	.	.	.23	.	.	?	.
43	0	0	.	.	.	.	.	.	.	.	?	460.67
44	0	0	.	.	.	.	.	.	.	.	?	854.38
45	0	0	.	.	.	.	.	.	.	.	?	722.29
46	0	0	.	.	.	.	.	.	.	.	?	667.91
47	0	0	1.60	.03	1.57	2.00	98.00	.	.	10.11	?	492.33
48	0	0	1.55	.02	1.53	1.00	99.00	.	.	.	?	518.01
49	0	0	1.00	.00	1.00	.00	100.00	.	.	.	?	353.39
50	0	0	1.73	.12	1.60	7.00	93.00	.	.	8.62	?	391.12
51	0	0	.00	.	.	.	.	.	.	.	?	812.14
52	0	0	.00	.	.	.	.	.	.	.	?	572.69
53	0	0	4.18	.08	4.09	2.00	98.00	.	.	.	?	.
54	0	0	5.20	.00	5.20	.00	100.00	.	.	.	605.65	633.50
55	0	0	6.20	.00	6.20	.00	100.00	.	.	.	640.68	737.73
56	1	0	4.00	.09	3.91	2.33	97.67	.15	.40	.	.	.
57	1	0	1.68	.04	1.64	2.33	97.67	.17	.54	.	.	.
58	1	0	1.23	.00	1.22	.33	99.67	.	.	.	.	1027.78
59	1	0	2.35	.02	2.33	1.00	99.00	.	.	.	.	818.91
60	1	0	1.33	.00	1.32	.33	99.67	.	.	.	.	.
61	1	0	1.95	.01	1.94	.67	99.33	.15	.42	6.86	.	665.58
62	1	0	3.88	.01	3.86	.33	99.67	.17	.46	25.35	?	?
63	1	0	1.68	.02	1.65	1.33	98.67	.17	.34	7.52	?	?
64	1	0	3.88	.01	3.87	.33	99.67	.22	.44	26.49	?	?
65	1	0	4.15	.22	3.93	5.33	94.67	.20	.49	31.51	?	?
66	1	0	3.80	.01	3.79	.33	99.67	.08	.39	5.41	?	?
67	.	.	.	.	.	.	.	.	.	.	?	?
68	.	.	.	.	.	.	.	.	.	.	.	.
69	1	0	2.10	.03	2.07	1.33	98.67	.12	.28	2.66	.	.
70	1	0	2.58	.01	2.57	.33	99.67	.12	.41	2.28	.	.
71	1	0	2.75	.02	2.73	.67	99.33	.18	.32	.	.	.
72	1	0	3.86	.01	3.85	.33	99.67	.22	.	.	.	.
73	1	0	3.93	.21	3.72	5.33	94.67	.20	.	.	.	.
74	1	0	1.75	.04	1.72	2.00	98.00	.	.	.	.	.
75	1	0	1.40	.00	1.40	.00	100.00	.	.	5.68	319.54	1166.18
76	1	0	3.30	.03	3.27	1.00	99.00	.	.	6.24	333.87	966.41
77	1	0	2.10	.00	2.10	.00	100.00	.	.	4.68	317.71	519.95
78	1	0	1.80	.04	1.76	2.00	98.00	.	.	.	.	1712.52

	mac.p38mapk	mac.Pp38mapk	CD163.percent	cd163.count	lung.injury.score	pmn.alv	pmn.interst	hyaline.mbms	prot.debris	septal.thickrg
40	.	.	.	.	.	.	.	.	?	?
41	.	.	.	.	.	.	.	.	.	.
42	.	.	.	.	.	.	.	.	.	.
43	.	.	.	.	.	.	.	.	.	.
44	.	.	.	.	.	.	.	.	.	.
45	.	.	.	.	.	.	.	.	.	.
46	.	.	.	.	.	.	.	.	.	.
47	.	.	21.81	.34	.	.	.	.	.	.
48	.	.	30.36	.47	.	.	.	.	.	.
49	.	.	48.26	.48	.	.	.	.	.	.
50	.	.	31.11	.50	.	.	.	.	.	.
51	.	.	.	.	.	.	.	.	.	.
52	.	.	.	.	.	.	.	.	.	.
53	.	.	.	.	.	.	.	.	.	.
54	.	.	.	.	.	.	.	.	.	.
55	.	.	.	.	.	.	.	.	.	.
56	.	.	.	.	14.0	.0	.0	1.0	1.0	.0
57	.	.	.	.	7.0	.0	.0	1.0	.0	.0
58	.	.	47.59	.58	30.0	.0	1.0	2.0	.0	1.0
59	.	.	22.42	.52	14.0	.0	.0	1.0	1.0	.0
60	.	.	19.74	.26	7.0	.0	.0	1.0	.0	.0
61	240.68	128.15	.	.	23.0	.0	1.0	1.0	.0	1.0
62	492.13	125.33	.	.	.	.	.	.	.	.
63	.	.	.	.	.	.	.	.	.	.
64	.	.	.	.	.	.	.	.	.	.
65	.	.	.	.	.	.	.	.	.	.
66	.	.	.	.	.	.	.	.	.	.
67	.	.	.	.	.	.	.	.	.	.
68	.	.	.	.	.	.	.	.	.	.
69	.	.	.	.	.	.	.	.	.	.
70	.	.	.	.	.	.	.	.	.	.
71	.	.	.	.	.	.	.	.	.	.
72	754.73	228.11	.	.	.	.	.	.	.	.
73	288.96	209.08	.	.	.	.	.	.	.	.
74	.	.	29.23	.50	.	.	.	.	.	.
75	.	.	54.91	.77	.	.	.	.	.	.
76	.	.	22.13	.72	.	.	.	.	.	.
77	.	.	48.90	1.03	.	.	.	.	.	.
78	.	.	48.62	.86	.	.	.	.	.	.

	Gln	IL1.LPS	cellcount	pmn.coun	macs.count	pmn.percent	macs.perc ent	prot	ldh	mac.ho1	mac.tnfa	mac.IL10
79	1	0	1.23	.02	1.20	2.00	98.00	.	.	.	.	276.53
80	1	0	2.35	.01	2.34	.30	99.70	.	.	.	.	612.37
81	?	?	?	?	?	?	?	?	?	?	473.04	.
82	?	?	?	?	?	?	?	.	.	.	519.10	404.50
83	1	0	5.50	.00	5.50	.00	100.00	.	.	.	541.74	.
84	0	1	66.25	64.93	1.33	98.00	2.00	.63	3.31	1.33	.	573.63
85	0	1	40.75	39.39	1.36	96.67	3.33	.40	1.93	4.61	.	279.80
86	0	1	21.38	20.38	.93	95.33	4.33	.97	2.28	.	.	609.33
87	0	1	.	.	.	.	.	.	.	.	.	801.17
88	0	1	.	.	.	.	.	.	.	.	.	678.88
89	0	1	97.00	92.15	4.85	95.00	5.00	2.59	5.27	.	.	653.21
90	0	1	180.25	174.25	6.00	96.67	3.33	1.59	4.12	.	.	1270.50
91	0	1	87.00	84.68	2.32	97.33	2.67	.70	2.18	.	.	.
92	0	1	65.00	63.70	1.30	98.00	2.00	.50	1.38	9.41	.	419.83
93	0	1	51.00	47.43	3.57	93.00	7.00	.74	1.54	8.48	.	.
94	0	1	70.00	64.23	5.78	91.75	8.25	.95	2.03	9.14	.	522.75
95	0	1	25.20	21.84	3.36	86.67	13.33	2.21	4.69	21.05	.	.
96	0	1	17.80	15.13	2.67	85.00	15.00	.90	3.21	16.74	.	.
97	0	1	34.00	30.15	3.85	88.67	11.33	.38	1.06	34.30	.	.
98	0	1	23.00	20.55	2.45	89.33	10.67	.41	1.05	42.67	.	.
99	0	1	63.80	51.04	12.76	80.00	20.00	4.15	.	.	.	.
100	0	1	14.50	11.99	2.51	82.67	17.33	2.31	.	.	.	.
101	?	?	?	?	?	?	?	?	?	?	.	.
102	?	?	?	?	?	?	?	?	?	.	.	.
103	0	1	28.90	28.61	.29	99.00	1.00	.75	3.83	8.48	.	.
104	0	1	42.00	40.32	1.68	96.00	4.00	2.03	6.54	9.14	.	.
105	0	1	43.50	43.50	.00	100.00	.00	.33	1.44	.	.	.
106	0	1	88.30	88.30	.00	100.00	.00	2.32	7.98	.	.	.
107	0	1	56.80	56.80	.00	100.00	.00	8.43	16.15	.	.	.
108	0	1	61.30	60.07	1.23	98.00	2.00	.73	2.23	.	.	.
109	0	1	12.30	12.30	.00	100.00	.00	3.79	13.96	.	.	.
110	0	1	49.30	48.46	.84	98.30	1.70	2.06	6.04	.	.	.
111	0	1	38.50	38.50	.00	100.00	.00	5.10	24.90	.	.	.
112	0	1	67.00	64.32	2.68	96.00	4.00	1.55	8.84	.	.	.
113	0	1	56.00	54.32	1.68	97.00	3.00	.64	5.02	.	.	.
114	0	1	115.00	106.95	8.05	93.00	7.00	.55	7.89	.	.	.
115	0	1	67.80	65.77	2.03	97.00	3.00	.	.	23.27	419.06	597.90
116	0	1	136.50	132.41	4.10	97.00	3.00	.	.	34.97	384.80	714.82
117	0	1	68.40	67.03	1.37	98.00	2.00	.	.	35.07	395.85	788.34

	mac.p38mapk	mac.Pp38mapk	CD163.percent	cd163.count	lung.injury.score	pmn.alv	pmn.interst	hyaline.mbms	prot.debris	septal.thickng
79	.	.	.	.	.	.	.	.	.	.
80	.	.	.	.	.	.	.	.	.	.
81	.	.	.	.	.	.	.	.	.	.
82	.	.	.	.	.	.	.	.	.	.
83	.	.	.	.	.	.	.	.	.	.
84	420.45	123.41	4.99	.07	61.0	2.0	.0	2.0	1.0	.0
85	352.48	144.17	4.41	.06	63.0	2.0	.0	2.0	1.0	1.0
86	683.78	179.11	3.24	.03	98.0	2.0	2.0	2.0	2.0	1.0
87	.	.	.	.	.	.	.	.	.	.
88	.	.	.	.	.	.	.	.	.	.
89	.	.	12.80	.62	54.0	2.0	.0	1.0	1.0	.0
90	.	.	26.13	1.57	29.0	1.0	.0	1.0	.0	1.0
91	.	.	17.97	.42	70.0	2.0	2.0	.0	.0	1.0
92	.	.	.	.	.	.	.	.	.	.
93	.	.	.	.	.	.	.	.	.	.
94	.	.	.	.	.	.	.	.	.	.
95	.	.	.	.	.	.	.	.	.	.
96	1213.80	259.20	.	.	.	.	.	.	.	.
97	.	.	.	.	.	.	.	.	.	.
98	.	.	.	.	.	.	.	.	.	.
99	.	.	.	.	.	.	.	.	.	.
100	.	.	.	.	.	.	.	.	.	.
101	.	.	.	.	.	.	.	.	.	.
102	.	.	.	.	.	.	.	.	.	.
103	.	.	.	.	.	.	.	.	.	.
104	.	.	.	.	.	.	.	.	.	.
105	.	.	.	.	.	.	.	.	.	.
106	.	.	.	.	.	.	.	.	.	.
107	.	.	.	.	.	.	.	.	.	.
108	.	.	.	.	.	.	.	.	.	.
109	.	.	.	.	.	.	.	.	.	.
110	.	.	.	.	.	.	.	.	.	.
111	.	.	.	.	.	.	.	.	.	.
112	.	.	.	.	.	.	.	.	.	.
113	.	.	.	.	.	.	.	.	.	.
114	.	.	.	.	.	.	.	.	.	.
115	.	.	37.30	.76	.	.	.	.	.	.
116	.	.	27.53	1.13	.	.	.	.	.	.
117	.	.	25.52	.35	.	.	.	.	.	.

	Gln	IL1.LPS	cellcount	pmn.coun	macs.count	pmn.percent	macs.perc ent	prot	ldh	mac.ho1	mac.tnfa	mac.IL10
118	0	1	205.00	202.95	2.05	99.00	1.00	.	.	.	?	?
119	0	1	188.00	172.96	15.04	92.00	8.00	.	.	.	501.70	932.57
120	1	1	74.00	64.14	9.86	86.67	13.33	.24	.21	.	.	.
121	1	1	54.80	52.06	2.74	95.00	5.00	.43	.82	.	.	.
122	1	1	93.10	80.69	12.41	86.67	13.33	.52	1.63	38.71	.	389.72
123	1	1	20.90	16.16	4.74	77.33	22.67	.30	.96	50.54	.	424.96
124	1	1	71.60	59.67	11.93	83.33	16.67	.63	2.96	51.08	.	490.78
125	1	1	34.20	26.56	7.64	77.67	22.33	.35	1.30	32.90	.	457.17
126	1	1	14.60	12.46	2.13	85.33	14.60	2.23	3.04	39.25	.	.
127	1	1	50.80	41.49	9.31	81.67	18.33	.90	2.44	6.90	.	.
128	1	1	46.30	37.81	8.49	81.67	18.33	2.41	4.31	25.08	.	.
129	1	1	29.50	26.35	3.15	89.33	10.67	.22	1.12	31.86	.	.
130	1	1	48.50	41.86	6.64	86.30	13.70	.	.	.	.	475.14
131	1	1	17.50	13.83	3.68	79.00	21.00	.39	1.42	17.47	.	761.26
132	1	1	48.50	41.87	6.63	86.33	13.67	.73	1.43	5.99	.	512.01
133	1	1	61.50	56.17	5.33	91.33	8.67	.71	.82	33.85	.	760.10
134	1	1	65.25	57.42	7.83	88.00	12.00	.98	1.46	.	.	452.04
135	1	1	12.63	8.29	4.33	65.67	34.33	.30	1.83	33.87	.	504.78
136	1	1	85.00	77.63	7.37	91.33	8.67	.95	2.52	.	.	.
137	1	1	87.75	79.85	7.90	91.00	9.00	1.81	3.02	.	.	.
138	1	1	71.00	63.42	7.58	89.33	10.67	.62	.99	.	?	?
139	1	1	111.80	97.63	14.17	87.33	12.67	.89	2.29	.	.	.
140	1	1	57.50	48.88	8.63	85.00	15.00	.	.	.	.	678.41
141	1	1	74.80	60.34	14.46	80.67	19.33	.	.	.	.	375.72
142	1	1	50.00	40.84	9.17	81.67	18.33	.	.	.	.	529.05
143	1	1	26.30	23.23	3.07	88.33	11.67	.	.	.	.	354.48
144	1	1	90.80	85.35	5.45	94.00	6.00	.	.	5.22	399.82	545.85
145	1	1	91.80	88.13	3.67	96.00	4.00	.	.	18.22	382.39	634.31
146	1	1	65.60	64.29	1.31	98.00	2.00	.	.	10.88	372.05	408.63
147	1	1	161.30	154.85	6.45	96.00	4.00	.	.	.	515.79	.
148	1	1	148.50	138.55	9.95	93.30	6.70	.	.	.	539.56	.
149	1	1	17.50	15.93	1.58	91.00	9.00	.	.	.	582.98	.
150	1	1	55.80	45.03	5.19	80.70	9.30	.	.	.	536.66	.

	mac.p38mapk	mac.Pp38mapk	CD163.percent	cd163.count	lung.injury.score	pmn.alv	pmn.interst	hyaline.mbms	prot.debris	septal.thickrg
118	.	.	.	.	.	.	.	.	.	.
119	.	.	.	.	.	.	.	.	.	.
120	.	.	.	.	75.0	2.0	1.0	1.0	2.0	.0
121	.	.	.	.	29.0	1.0	.0	1.0	.0	1.0
122	.	.	.	.	84.0	2.0	2.0	2.0	.0	1.0
123	3016.68	255.16	.	.	68.0	2.0	1.0	1.0	1.0	.0
124	2594.00	204.38	.	.	44.0	2.0	.0	.0	.0	2.0
125	3400.72	234.02	.	.	100.0	2.0	2.0	2.0	2.0	2.0
126	918.89	205.98	.	.	.	.	.	.	.	.
127	.	.	.	.	.	.	.	.	.	.
128	.	.	.	.	.	.	.	.	.	.
129	.	.	.	.	.	.	.	.	.	.
130	.	.	.	.	.	.	.	.	.	.
131	.	.	.	.	.	.	.	.	.	.
132	.	.	.	.	.	.	.	.	.	.
133	.	.	.	.	.	.	.	.	.	.
134	.	.	.	.	.	.	.	.	.	.
135	.	.	.	.	.	.	.	.	.	.
136	.	.	74.60	5.50	.	.	.	.	.	.
137	.	.	55.77	4.40	.	.	.	.	.	.
138	.	.	63.06	4.78	.	.	.	.	.	.
139	.	.	53.32	7.55	.	.	.	.	.	.
140	.	.	13.87	1.20	.	.	.	.	.	.
141	.	.	12.10	1.75	.	.	.	.	.	.
142	.	.	16.38	1.50	.	.	.	.	.	.
143	.	.	22.47	.69	.	.	.	.	.	.
144	.	.	22.04	1.20	.	.	.	.	.	.
145	.	.	20.72	.76	.	.	.	.	.	.
146	.	.	21.47	.28	.	.	.	.	.	.
147	.	.	.	.	.	.	.	.	.	.
148	.	.	.	.	.	.	.	.	.	.
149	.	.	.	.	.	.	.	.	.	.
150	.	.	.	.	.	.	.	.	.	.
